# Dislocation-Loop-Induced Secondary Recrystallization in Fe-3 wt.% Si: Y-Stabilized 〈100〉 Loops as Inhibitor Alternatives

**DOI:** 10.3390/ma19194152

**Published:** 2026-09-29

**Authors:** Shun Wang, Weixue Dou, Li Xie, Hongguo Wang, Shuo Yang, Yang Tang

**Affiliations:** 1 Postdoctoral Research Station of Jingye Group, Jingye Group Research Institute, Shijiazhuang 050409, China; yangshuo0224@yeah.net; 2 Hebei Jingye Wide Plate Technology Co., Ltd., Shijiazhuang 050409, China; 3 Postdoctoral Research Station, Northeastern University, Shenyang 110819, China; hitdianqitangyang@163.com; 4 Jingye Steel Group Co., Ltd., Shijiazhuang 050409, China; xieli_20031710@163.com (L.X.); whghongguo@163.com (H.W.)

**Keywords:** grain-oriented silicon steel, 〈100〉 dislocation loops, loop-induced inhibition mechanism, rare-earth segregation, Goss texture, grain-boundary pinning

## Abstract

Conventional grain-oriented silicon steel (GO) relies on AlN/MnS-type inhibitors and long purification anneals, which increase alloy design complexity, energy consumption and cost. This study establishes a dislocation-loop-induced secondary recrystallization route in Fe-3 wt.% Si grain-oriented silicon steel produced by twin-roll strip casting with rare-earth Y microalloying. Steels containing 0, 0.05 and 0.1 wt.% Y were cold-rolled to 0.30 mm and subjected to primary and secondary recrystallization annealing. Electron backscatter diffraction (EBSD) reveals that 0.05 wt.% Y promotes {100}-oriented columnar solidification grains and strengthens the surface {111}〈110〉/{111}〈112〉 γ-fiber after rolling, increasing shear-band density and Goss nucleation sites while refining the primary-recrystallized matrix. Transmission electron microscopy shows abundant, thermally stable 〈100〉 dislocation loops (~50–100 nm) in Y-containing steels, rather than the commonly reported 1/2〈111〉 loops. These 〈100〉 loops act as effective grain-boundary pinning centers, suppressing normal grain growth and triggering abnormal Goss growth during high-temperature annealing. The 0.05 wt.% Y steel attains a secondary-recrystallization fraction of ~70%, with B_8_ = 1.71 T and P_1.7_/_50_ = 2.81 W·kg^−1^, whereas the Y-free alloy exhibits only normal grain growth and a weak Goss texture; excessive Y (0.1 wt.%) over-suppresses boundary mobility. The strong Y–vacancy affinity rationalizes the formation, thermal stability and potential recyclability of 〈100〉 loops, providing an inhibitor-free, energy-efficient pathway for manufacturing high-performance grain-oriented silicon steel.

## 1. Introduction

Grain-oriented silicon steel, a key soft-magnetic material for transformer and large-generator stator cores, traditionally employs second-phase particles such as MnS and AlN as inhibitors to induce secondary recrystallization [1]. The use of such inhibitors renders manufacturing highly sensitive to composition and processing, requiring tight elemental tolerances and multi-step thermal schedules. In particular, compositional fluctuations of Al and N that control inhibitor formation must be limited to within 10 ppm [2]. Relative to non-oriented grades, additional steps—normalization, decarburization, and nitriding—are required to obtain fine, dispersed precipitates. Even after secondary-recrystallization annealing, a prolonged high-temperature purification anneal is commonly applied to decompose inhibitors and remove N, S, and other elements under a strongly reducing atmosphere. Industrially, the cumulative time spent at elevated temperature exceeds 100 h for grain-oriented silicon steel production [3].

With improved understanding of dislocation characteristics, dislocation engineering has emerged as an approach that introduces and stabilizes high densities of mobile dislocations to achieve synergistic strengthening and ductility, providing an alternative to conventional alloying strategies. By controlling the dislocation density and substructure, multiple strengthening mechanisms and deformation modes can operate concurrently, enabling alloys with ultrahigh strength and excellent ductility. As a fundamental microstructural defect in metals, dislocations strongly influence mechanical, electromagnetic, and related properties. For example, Gutierrez-Urrutia et al. rationalized the strength–ductility synergy in Fe-30.5Mn-2.1Al-1.2C (wt.%) steel through dislocation-substructure refinement and twin activation under tension [4]. He and co-workers integrated dislocation engineering with room-temperature quenching and partitioning: dislocations introduced by warm rolling impeded transformation interface motion and refined martensite blocks, yielding high strength and toughness [5]. In medium-Mn steels, multi-pass rolling produced extremely high dislocation densities that persisted through tempering, accumulating near grain boundaries and interacting during deformation to enhance both yield strength and ductility; warm rolling was further used to stabilize retained austenite during room-temperature deformation [6].

It is noteworthy that dislocation loops, as a particular type of dislocation, are generally generated by dislocation multiplication or vacancy clustering. However, studies on the control of dislocation loops remain limited and have mainly focused on their formation and evolution during irradiation. Since the introduction of dislocation loops often causes swelling and hardening, most research has aimed at eliminating them through nanoscale precipitation. In BCC iron, the typical types of dislocation loops are 1/2〈111〉 and 〈100〉. Over the past decades, extensive research has been conducted on the migration behavior of 1/2〈111〉 loops. N. Gaoa reported that due to their strong pinning effect, 〈100〉 loops are usually regarded as obstacles similar to inhibitors in materials [7]. The team of Mingxin Huang at the University of Hong Kong [8,9] successfully produced steels with ultrahigh strength and excellent ductility by replacing alloying with high-density mobile dislocations, enabling synergistic operation of multiple strengthening mechanisms and deformation modes. Compared with line dislocations, dislocation loops possess distinct formation pathways, evolution behavior, and influences on macroscopic properties. In metallic materials, dislocation loops are mainly generated during quenching, deformation, and irradiation processes [9,10,11,12]. For BCC steels, two Burgers vector types—1/2〈111〉 and 〈100〉—are commonly observed [13]. Wirth et al. [14], using molecular dynamics based on the Finnis–Sinclair potential, calculated that the migration barriers of 1/2〈111〉 prismatic loops remain below 0.1 eV from 100 to 900 K, indicating strong diffusivity and rapid one-dimensional glide along the Burgers vector direction; this was experimentally verified by Osetsky et al. [15]. Marian et al. [16] systematically investigated the formation and growth of 〈100〉 interstitial loops in ferritic materials and calculated their migration energy to be approximately 2.5 eV, far higher than that of 1/2〈111〉 loops. Thus, 〈100〉 loops exhibit low mobility and are typically regarded as obstacles analogous to precipitates [17,18]. Lehtinen et al. [19] compared the strengthening effects of precipitates and 1/2〈111〉 loops in irradiated materials through accelerated molecular dynamics, showing that although the mechanisms differ, precipitates and loops with similar density and size contribute comparably to yield strength.

Motivated by these insights, this work explores whether high-density dislocation loops can function as inhibitors to induce secondary recrystallization in grain-oriented silicon steel, thereby eliminating the purification-annealing step, reducing energy use, and simplifying composition control. Specifically, we examine Fe-3 wt.% Si processed without conventional inhibitors, where rare-earth yttrium and sub-rapid solidification are combined to generate dense loops whose morphology and rare-earth segregation reduce mobility and enhance thermal stability, enabling dislocation-loop-induced secondary recrystallization. Compared with conventional inhibitor-based processing, the proposed route obviates long purification anneals and markedly simplifies high-temperature schedules; relative to other inhibitor-free approaches, loop-induced secondary recrystallization proceeds more rapidly and is expected to complete in a shorter time.

## 2. Experimental Materials and Methods

### 2.1. Materials and Processing

For comparative purposes, three ultra–low-carbon (C ≤ 50 ppm, mass fraction) grain-oriented silicon steel strips with different Y contents were prepared by the twin-roll strip casting process. The Si content was controlled within 3.00–3.20% (mass fraction), and the Y contents were 0, 0.05%, and 0.1% (mass fraction), denoted as samples A, B, and C, respectively. Their chemical compositions are listed in Table 1. The twin-roll strip casting experiment was performed in the DSL Laboratory of Northeastern University. Experimental raw materials were charged into a 50 kg vacuum medium-frequency induction furnace (Twin-Roll Strip Casting Machine, Northeastern University, Shenyang, China) for heating; subsequent to melting, the raw materials underwent refining and alloying treatments. The molten steel was guided to a tundish preheated to approximately 1200 °C via a casting nozzle. Thereafter, the molten steel in the tundish flowed through a nozzle into the molten pool formed by crystallization rolls and side dams, and was ultimately rapidly solidified into a 2 mm-thick cast strip. To prevent molten steel oxidation, both smelting and casting processes were conducted under inert gas protection. In this experiment, the casting temperature was controlled within the range of 1500~1530 °C, ensuring a casting superheat of 25~35 °C. The rolling and heat-treatment procedure consisted of single-stage cold rolling followed by primary recrystallization annealing and secondary recrystallization annealing. (1) Cold rolling: the as-cast strips (150 mm × 100 mm × 2 mm) were directly cold-rolled to 0.30 mm, with a reduction of approximately 85%. (2) Primary recrystallization annealing: the cold-rolled sheets were annealed at 850 °C for 5 min. (3) Secondary recrystallization annealing: 850 °C heating to 1200 °C at 20 °C·h^−1^ in a protective atmosphere of H_2_:N_2_ = 3:1 and held for 2 h. Before annealing, an MgO coating was applied as a separator to prevent oxidation and sticking.

### 2.2. Characterization and Testing Methods

The Microstructure and crystallographic orientation were characterized by Electron Backscattered Diffraction (EBSD) detector attached to a field-emission Scanning Electron Microscope (SEM, Model: Zeiss Ultra 55), manufactured by Carl Zeiss Microscopy GmbH, Oberkochen, Germany. Data cleaning and orientation analysis were carried out using HKL Channel 5, Version 5.1, Oxford Instruments (HKL Technology), Hobro, Denmark, adopting a tolerance angle of 15° for accurate orientation determination. Specimens were cut from the as-cast strips and annealed sheets (10 mm along the rolling direction (RD) × 8 mm) and electropolished in a C_2_H_5_OH:HClO_4_:H_2_O (13:2:1, vol.%) solution at 20–25 V for 18–25 s to expose the RD–normal direction (ND) plane for EBSD mapping.

Macrostructure and texture: Macrostructures on the RD–transverse direction (TD) plane after secondary recrystallization were recorded after removal of the forsterite glass film in dilute HCl, which enabled clear observation of millimeter-sized grains. Macrotexture was measured using a Bruker D8 Discover X-ray (XRD, D8 Advance, Bruker AXS GmbH, Karlsruhe, Germany) diffractometer with Co Kα radiation by acquiring {110}, {200}, and {112} pole figures, from which the orientation distribution function (ODF, L_max = 22) was calculated. Rectangular specimens (20 mm × 18 mm) were stress-relief etched in 10% HCl, and surface (0H) and mid-thickness (1/2H) layers were distinguished.

TEM/EDS: A FEI Tecnai G2 F20 transmission electron microscope (Thermo Fisher Scientific (FEI Company), Hillsboro, OR, USA) was employed to examine precipitates and dislocations, including dislocation loops. Thin foils were prepared by wire electrical discharge machining followed by mechanical grinding to <50 μm and twin-jet electropolishing in a solution of 12.5 vol.% HClO_4_ + 87.5 vol.% C_2_H_5_OH. The composition and spatial distribution of precipitates were analyzed by TEM–energy-dispersive spectroscopy (TEM–EDS).

Magnetic properties: Magnetic loss P_1.7_/_50_ was measured at 1.7 T and 50 Hz, and magnetic induction B_8_ at 800 A·m^−1^ and 50 Hz, using a Automatic Magnetic-Property Testing System (MATS-2010M, Hunan Lianzhong Technology Co., Ltd., Changsha, China) The specimens for magnetic measurements had dimensions of 100 mm × 30 mm (RD–TD). Each magnetic value represents the average of at least three specimens cut from different positions of the same strip.

## 3. Results

### 3.1. Evolution of Solidification Microstructure and Initial Texture in As-Cast Fe-3 wt.% Si Strips

Figure 1 shows the microstructure and texture evolution of as-cast Fe-3 wt.% Si strips with different Y contents. The as-cast microstructures reveal that Strip A (0 wt.% Y) exhibits predominantly coarse equiaxed grains at the surface with a few columnar grains, whereas the strip center consists of finer equiaxed grains (average grain size ≈ 352 μm). With the addition of Y, the microstructure changes to elongated {100}-oriented columnar grains; in the 0.10 wt.% Y alloy, these columns are finer and more densely packed than in the 0.05 wt.% Y alloy, consistent with grain-boundary segregation of Y suppressing coarsening. The ODF sections reveal an almost random orientation distribution for Strip A, while Strips B and C develop a pronounced {100} λ-fiber that correlates with the columnar morphology. This columnar {100} texture provides favorable initial conditions for the stronger surface {111}〈110〉 component observed after rolling and increases the number of potential shear-band sites available for Goss nucleation.

### 3.2. Cold-Rolled Microstructure and Macrotexture

The microstructures of the three steels after single-stage cold rolling to 0.30 mm are shown in Figure 2a–c. Steels A and B exhibit typical elongated deformation grains containing micro-deformation bands oriented at approximately 15–40° to the rolling direction (RD). In contrast, Steel C contains fewer intragranular deformation features, and the micro-deformation bands are less pronounced.

Figure 2d–i show the macrotextures of the surface and center layers of the cold-rolled sheets. The surface textures are dominated by α- and γ-fiber components, with the strongest γ-fiber corresponding to the {111}〈110〉 texture. The orientation densities of this {111}〈110〉 component are 2.56, 4.43, and 3.48 for Steels A, B, and C, respectively. Thus, the addition of Y enhances the surface {111}〈110〉 component, with Steel B exhibiting the highest intensity. This behavior is attributed to the columnar grains formed in the Y-containing as-cast strips, which undergo collective rotation during cold rolling, thereby promoting crystal rotation and strengthening the {111}〈110〉 component. In contrast, Steel A consists mainly of equiaxed grains that deform more uniformly and rotate more slowly, resulting in a weaker {111}〈110〉 texture. In Steel C, the higher Y content further impedes dislocation motion during deformation, limiting the enhancement of the surface {111}〈110〉 component; consequently, the strongest surface texture is obtained in the Fe-3 wt.% Si–0.05 wt.% Y alloy.

As shown in Figure 2g–i, the dominant texture components in the center layers are also α- and γ-fibers, with the strong γ-fiber again corresponding to the {111}〈110〉 texture. The orientation densities are 6.97, 3.98, and 3.02 for Steels A, B, and C, respectively, which is opposite to the trend observed at the surface. The {111}〈110〉 texture in the center layer weakens with Y addition because this region lies at the transition between equiaxed and columnar grains, where grain morphology and stress distribution exert a weaker influence on grain rotation. In Steel C, the higher Y content further suppresses crystal rotation, leading to a more pronounced reduction in the {111}〈110〉 intensity.

### 3.3. Primary Recrystallization Microstructure and Texture

Figure 3 shows the orientation imaging maps and the corresponding ODF sections at φ_2_ = 45° for the three steels after single-stage cold rolling followed by primary recrystallization annealing. The Fe-3 wt.% Si–0.05 wt.% Y and Fe-3 wt.% Si–0.10 wt.% Y alloys develop fine and uniform primary-recrystallized microstructures with average grain sizes of 11.6 and 12.0 μm, respectively. In contrast, the Fe-3 wt.% Si alloy exhibits a coarser recrystallized structure with an average grain size of 15.8 μm. After primary annealing, all three steels display typical γ-type recrystallization textures.

Figure 3d–i present the macrotextures of the surface and center layers after primary recrystallization. As shown in Figure 3d–f, the dominant surface texture after primary annealing is the {111}〈112〉 component within the γ-fiber, and its orientation density decreases in the order Fe-3 wt.% Si–0.05 wt.% Y (5.28) > Fe-3 wt.% Si–0.10 wt.% Y (3.30) > Fe-3 wt.% Si (2.76). This trend is associated with the inheritance of the {111}〈110〉 component formed during cold rolling. The surface {111}〈110〉 texture is stronger in Steel B than in Steel C, and this component tends to transform into {111}〈112〉 during primary annealing. Consequently, the {111}〈112〉 texture is more strongly enhanced in Steel B than in Steel C. As shown in Figure 3g–i, the intensity of the {111}〈112〉 texture in the center layer of Steel B is lower than that in Steel A. This is because Steel A develops a strong {111}〈112〉 texture in the center layer during cold rolling, which is largely inherited during primary annealing and thus retains a high intensity.

In contrast, the pronounced enhancement of the {111}〈112〉 texture in Steel C can be rationalized as follows. The {111}〈112〉 component has a strong nucleation advantage but a limited growth advantage and is therefore easily consumed by grains with other orientations. In Steel C, the presence of rare-earth Y atoms significantly retards grain growth and suppresses the consumption of {111}〈112〉-oriented grains, preserving their nucleation advantage. As a result, a pronounced increase in the {111}〈112〉 texture is observed.

### 3.4. Secondary Recrystallization

Figure 4 reveals distinct secondary recrystallization behavior among the three steels. The Y-free alloy (A) exhibits normal grain growth, indicating insufficient inhibition to promote selective Goss growth. In contrast, the 0.05 wt.% Y steel (B) undergoes pronounced secondary recrystallization, characterized by a sharp grain-boundary migration front and preferential abnormal growth of Goss-oriented grains. The resulting sharp Goss texture enhances the magnetic properties, yielding B_8_ = 1.71 T and P_1.7_/_50_ = 2.81 W·kg^−1^. At the higher Y level of 0.10 wt.% (Steel C), grain coarsening is strongly suppressed and only partial abnormal growth is observed. Over-precipitate of Y is expected to reduce grain-boundary mobility and hinder the completion of Goss-oriented growth consistent with an optimal inhibition balance being achieved at 0.05 wt.% Y.

## 4. Discussion

### 4.1. Shear-Band-Assisted Goss Nucleation

Figure 5 illustrates the early stage of selective Goss development. In Strip A, isolated Goss nuclei are sparse and are mostly separated from shear-band intersections. Strip B exhibits a markedly higher Goss fraction: green Goss grains are concentrated along internal shear bands embedded in {111}〈110〉/{111}〈112〉 deformed matrices, indicating shear-band-assisted nucleation and early growth. In Strip C, although shear bands are still present, their continuity is reduced and Cube-oriented regions expand, thereby limiting further increase in the Goss fraction. These observations indicate that a Y addition of 0.05 wt.% increases shear-band density by retarding dislocation glide, thereby providing more effective nucleation sites for Goss. In contrast, excess Y (0.10 wt.%) refines the initial solidification structure and disrupts band continuity, which is unfavorable for sustained Goss development.

As shown in Figure 6, sub-rapid solidification produces Y-containing precipitates segregated along grain boundaries, primarily Y_2_O_3_ and Fe–Y intermetallic phases. The formation of these thermally stable oxides and intermetallics reflects the high oxygen affinity and limited solubility of Y in Fe–Si melts. Their boundary-segregated distribution provides potential heterogeneous nucleation sites for dislocation loops during subsequent annealing. These Y-rich regions locally reduce grain-boundary mobility, thereby enhancing pinning efficiency and contributing to the stability of the 〈100〉 dislocation loops observed in the primary-recrystallized matrix.

According to the TEM observations in Figure 7a, a large number of dislocation loops are distributed within the primary-recrystallized matrix, with sizes comparable to those of conventional inhibitors (≈50–100 nm). When the incident beam is tilted to the [001] zone axis, the habit-plane normal of the loops becomes perpendicular to the beam, and the loops appear in an edge-on configuration (Figure 7b). Based on extinction rules and the crystallographic relationship between the habit plane and the projection plane [20], the Burgers vector of these loops is identified as 〈100〉.

Combined with the secondary recrystallization results in Figure 4b, it can be concluded that the dislocation loops formed during primary recrystallization in Fe-3 wt.% Si–0.05 wt.% Y promote abnormal Goss grain growth during high-temperature annealing. In contrast, the Fe-3 wt.% Si alloy without Y does not form dislocation loops under the same processing conditions, and its grains undergo normal growth without secondary recrystallization (Figure 4a).

It is noteworthy that the occurrence of 〈100〉 dislocation loops in body-centered cubic (BCC) metals is not intuitive. Because the close-packed direction in BCC crystals is 〈111〉, only 1/2〈111〉 dislocation loops would typically be expected. Moreover, the energy of 〈100〉 loops is higher than that of 1/2〈111〉 loops (as the loop energy scales with the square of the Burgers vector), making their formation energetically unfavorable. Consequently, the origin of 〈100〉 loops has long been a topic of interest in nuclear materials research [21]. Eyre et al. [22] proposed that self-interstitial atoms (SIAs) generated under irradiation first cluster on close-packed {110} planes to form 1/2〈110〉 stacking faults, which then transform into 1/2〈111〉 and 〈100〉 dislocation loops through shear reactions. Master et al. [23] suggested that 〈100〉 loops may also form through direct reactions between two 1/2〈111〉 loops. Later, Marian et al. [16] argued that only two 1/2〈111〉 loops of comparable size can react to form a 〈100〉 loop, with the initial habit plane being {110}, which subsequently transforms into {100} by atomic rearrangement or rotation mechanisms.

However, the formation and evolution of dislocation loops under irradiation are highly complex. In situ TEM studies by Ran Guang’s group at Xiamen University [24,25] showed that strict size equivalence is not required for two 1/2〈111〉 loops to react and form a 〈100〉 loop. Irradiation can directly generate 〈100〉 loops or promote the transformation of 1/2〈111〉 loops into 〈100〉-type loops. In addition, the evolution of dislocation loops depends strongly on irradiation dose and temperature. Dudarev et al. [26] demonstrated by theoretical calculations that 1/2〈111〉 loops are more stable at low temperatures, whereas 〈100〉 loops dominate at high temperatures. Numerous studies have also reported [27] that increasing irradiation dose enlarges loop size and increases the fraction of 〈100〉 dislocation loops. When the temperature is further raised to ~550 °C, 〈100〉 loops may decompose into more mobile 1/2〈111〉 loops, which are subsequently absorbed by grain boundaries and other interfaces [28].

In summary, the formation mechanism of 〈100〉 dislocation loops remains unresolved, and most existing studies have focused on their behavior under irradiation rather than under solidification or thermomechanical conditions.

The 〈100〉 dislocation loops observed in grain-oriented silicon steel in this study differ markedly from those reported previously. The schematic diagram of the mechanism by which dislocation loops induce secondary recrystallization in this study is shown in Figure 8. In particular, these 〈100〉 loops remain stable even during high-temperature annealing. At elevated temperatures, strong lattice vibrations generate a high density of vacancies in the alloy matrix. During slow cooling, vacancies migrate rapidly and are readily absorbed by grain boundaries, leading to a substantial reduction in vacancy concentration. By contrast, during rapid cooling a large number of vacancies are retained to room temperature and agglomerate into vacancy clusters, which subsequently collapse into dislocation loops. Such dislocation loops may further trigger processes such as spinodal decomposition and second-phase precipitation, thereby influencing the macroscopic properties of the material.

Rare-earth elements have strong affinity for vacancies. By adding rare-earth yttrium to inhibitor-free grain-oriented silicon steel and combining it with sub-rapid solidification, a high density of dislocation loops can be obtained. TEM characterization further revealed that these dislocation loops do not possess the commonly observed 1/2〈111〉 Burgers vector, but instead exhibit the high-temperature-stable 〈100〉 Burgers vector. Previous studies have shown that 〈100〉 dislocation loops in BCC metals behave similarly to second-phase particles and can effectively hinder grain growth.

More importantly, the formation of dislocation loops is closely associated with the presence of rare-earth yttrium. Dislocation loops and their complexes with Y formed at low temperatures gradually decompose upon slow heating as the solubility of Y increases. During subsequent cooling, the loops can reform. Therefore, compared with conventional inhibitors used in grain-oriented silicon steel, dislocation loops are recyclable, offering significant potential for application in the production of ultra-thin grain-oriented silicon steel.

The key to inducing secondary recrystallization in grain-oriented silicon steel is the suppression of normal grain growth during primary recrystallization. Unlike conventional inhibitors, dislocation loops act as two-dimensional defects, and their interactions with grain boundaries are governed by unique mechanisms. The Burgers vector of the loop, its inclination with respect to the boundary, and the degree of rare-earth precipitate all influence the physical processes governing loop–boundary interactions.

Han Weizhong’s group at Xi’an Jiaotong University [29] systematically summarized the reactions between interstitial-type dislocation loops and coherent twin boundaries. Interstitial loops generated by irradiation merge into the coherent twin boundary upon impact and form nanoscale protrusions. These bowed-out twin boundaries absorb nearby vacancy clusters produced by irradiation, and subsequent recombination of vacancies and interstitial atoms restores the boundary to a flat configuration, achieving self-healing. Aragon et al. [30] simulated, at the atomic scale, the interaction between dislocation loops and high-angle boundaries in an FCC matrix. High-angle boundaries can absorb dislocation loops, mitigating stress concentrations caused by dislocation pile-up while simultaneously altering the atomic structure of the grain boundary.

It should be noted that existing studies primarily focus on the absorption of dislocation loops by grain boundaries and do not fully address the pinning effect exerted by dislocation loops on boundaries. Moreover, the 〈100〉 dislocation loops identified in this study exhibit exceptional thermal stability. Therefore, elucidating the interaction mechanism and the unique pinning behavior of these highly stable loops represents another key issue in this work.

### 4.2. Overall Mechanism of Dislocation-Loop-Induced Secondary Recrystallization

The present results demonstrate that secondary recrystallization in Fe-3 wt.% Si strip-cast steel can be induced by high-density 〈100〉 dislocation loops rather than by conventional precipitate inhibitors. Sub-rapid solidification of the Y-containing melt produces boundary-segregated Y_2_O_3_ and Fe–Y intermetallic phases, which act as efficient vacancy traps and heterogeneous nucleation sites for dislocation loops during subsequent thermomechanical processing. Under the combined effects of vacancy supersaturation and strong Y–vacancy affinity, nanoscale 〈100〉 dislocation loops with sizes comparable to classical inhibitors (≈50–100 nm) are formed and retained in the primary-recrystallized matrix.

These loops operate as effective two-dimensional pinning centers during high-temperature annealing. In the Fe-3 wt.% Si–0.05 wt.% Y alloy, the 〈100〉 loops, together with enhanced surface {111}〈110〉/{111}〈112〉 γ-fiber textures and shear-band networks, selectively retard the growth of non-Goss matrix grains while allowing Goss-oriented grains to grow abnormally. The resulting sharp Goss texture leads to a high secondary-recrystallization fraction and improved magnetic properties. When the Y content is too low, dislocation loops are absent and only normal grain growth occurs; when the Y content is excessive, over-segregation reduces boundary mobility and disrupts shear-band continuity, so that Goss development remains incomplete. These observations indicate that dislocation-loop-induced secondary recrystallization is highly sensitive to the balance between loop density, boundary mobility, and stored-energy distribution.

A further feature of the present mechanism is the intrinsic recyclability of the dislocation loops. Dislocation loops and their complexes with Y decompose during slow heating as the solubility of Y increases, and can reform upon cooling as vacancies are recaptured, in contrast to precipitate inhibitors that coarsen irreversibly. This recyclability, combined with the strong thermal stability of 〈100〉 loops, provides a flexible means of tuning inhibition strength over multiple thermal cycles. Overall, by exploiting rare-earth-assisted vacancy trapping and strip-casting sub-rapid solidification, this work establishes a dislocation-loop-based inhibition strategy that enables secondary recrystallization without long purification anneals, offering a low-cost, low-alloying and energy-efficient route for manufacturing high-performance grain-oriented silicon steel.

## 5. Conclusions

A dislocation-loop-induced secondary recrystallization route was established for Fe-3 wt.% Si grain-oriented silicon steel produced by twin-roll strip casting with rare-earth Y microalloying. The main conclusions are as follows:

(1) Role of Y in solidification, deformation and primary recrystallization. The addition of 0.05 wt.% Y promotes the formation of {100}-oriented columnar grains during solidification and strengthens the surface {111}〈110〉/{111}〈112〉 γ-fiber after cold rolling, thereby increasing shear-band density and Goss nucleation sites. After primary recrystallization, the Y-containing steels exhibit finer and more homogeneous grains than the Y-free alloy, with a pronounced surface {111}〈112〉 texture inherited from the deformation γ-fiber.
(2) Formation and function of 〈100〉 dislocation loops. Sub-rapid solidification combined with Y microalloying generates abundant, thermally stable 〈100〉 dislocation loops (~50–100 nm) in the primary-recrystallized matrix, instead of the commonly reported 1/2〈111〉 loops. These 〈100〉 loops act as effective two-dimensional grain-boundary pinning centers, functionally analogous to precipitate inhibitors, suppressing normal grain growth and enabling abnormal Goss growth during high-temperature annealing.
(3) Optimal Y content window and magnetic performance. The steel containing 0.05 wt.% Y achieves a secondary-recrystallization fraction of ~70%, a sharp Goss texture and improved magnetic properties (B_8_ = 1.71 T, P_1.7_/_50_ = 2.81 W·kg^−1^). In contrast, the Y-free alloy exhibits only normal grain growth with a weak Goss component, whereas excessive Y (0.1 wt.%) over-suppresses boundary mobility and disrupts shear-band continuity, resulting in incomplete abnormal Goss growth. These results suggest a relatively narrow Y content window for effective dislocation-loop-based inhibition.
(4) Mechanistic and processing implications. The strong affinity between Y and vacancies rationalizes the formation, thermal stability and potential recyclability of 〈100〉 loops via Y–vacancy complexes. Y-stabilized 〈100〉 dislocation loops therefore provide a viable alternative to conventional inhibitors, enabling secondary recrystallization without long-duration purification annealing. This offers an inhibitor-free, simplified and energy-efficient processing route for grain-oriented silicon steel and establishes a composition–process–microstructure–property framework for dislocation-loop-controlled secondary recrystallization.

## Figures and Tables

**Figure 1:**
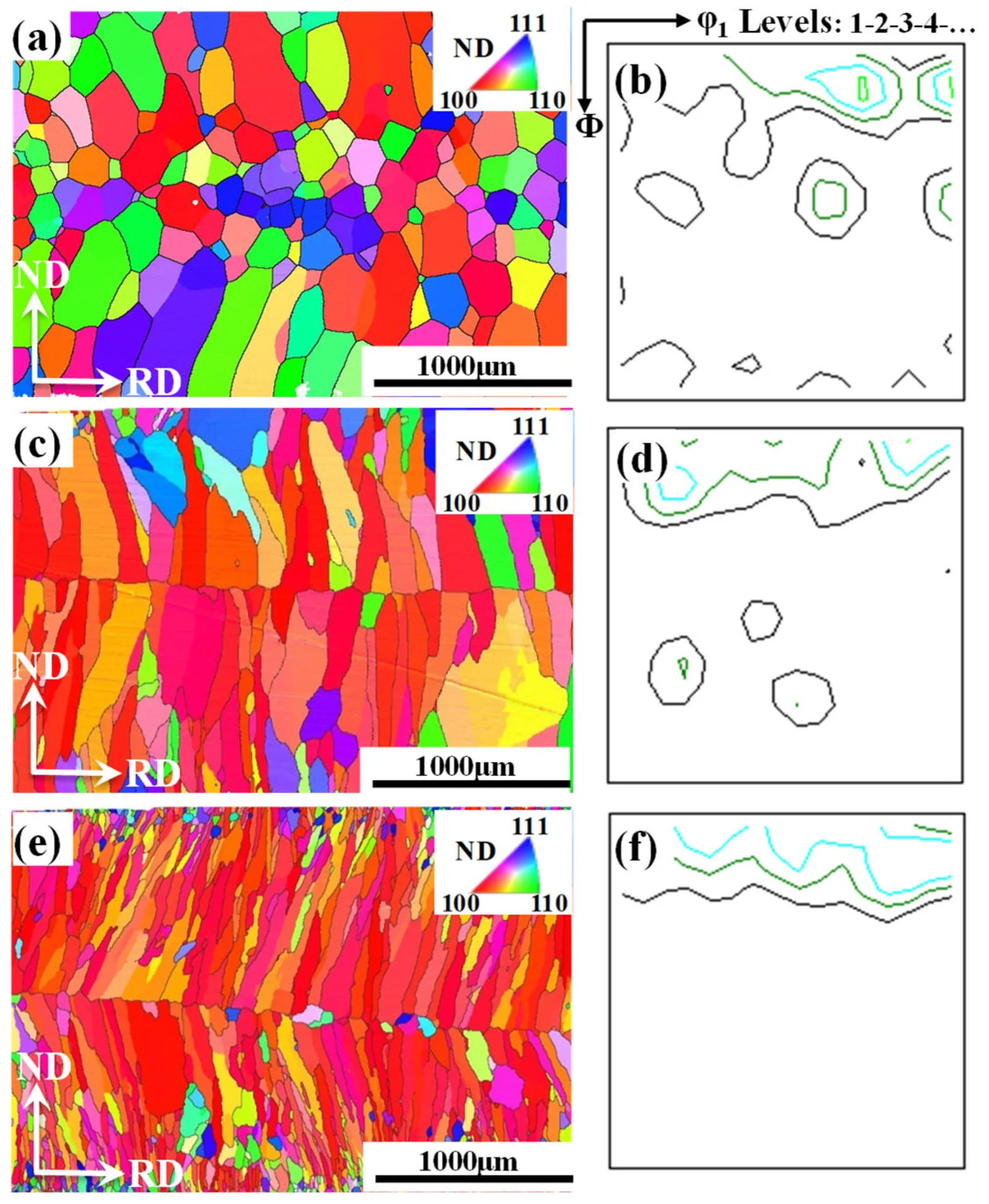
Microstructure and texture evolution of as-cast Fe-3 wt.% Si strips with different Y contents. (**a**,**b**) Strip A (0 wt.%Y); (**c**,**d**) Strip B (0.05 wt.% Y); (**e**,**f**) Strip C (0.1 wt.% Y). (**a**,**c**,**e**) Orientation imaging maps; (**b**,**d**,**f**) ODF sections at φ_2_ = 45°.

**Figure 2:**
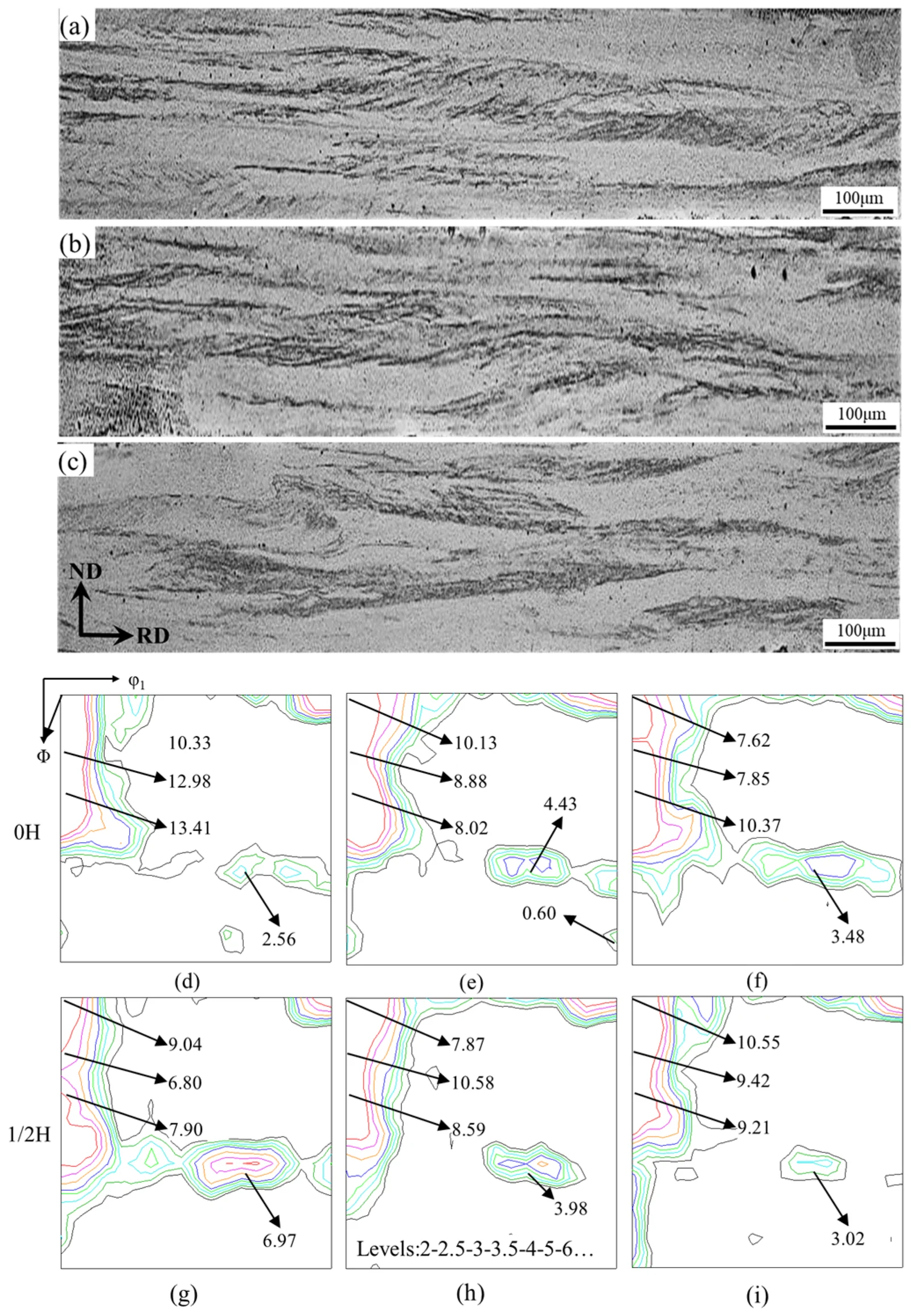
Microstructure and macrotexture of the cold-rolled sheets (t = 0.30 mm). (**a**–**c**) Surface microstructures after single-stage cold rolling for Strips A (0 wt% Y), B (0.05 wt% Y) and C (0.1 wt% Y); (**d**–**f**) Surface (0H) φ_2_ = 45° ODF sections; (**g**–**i**) mid-thickness (1/2H) φ_2_ = 45° ODF sections.

**Figure 3:**
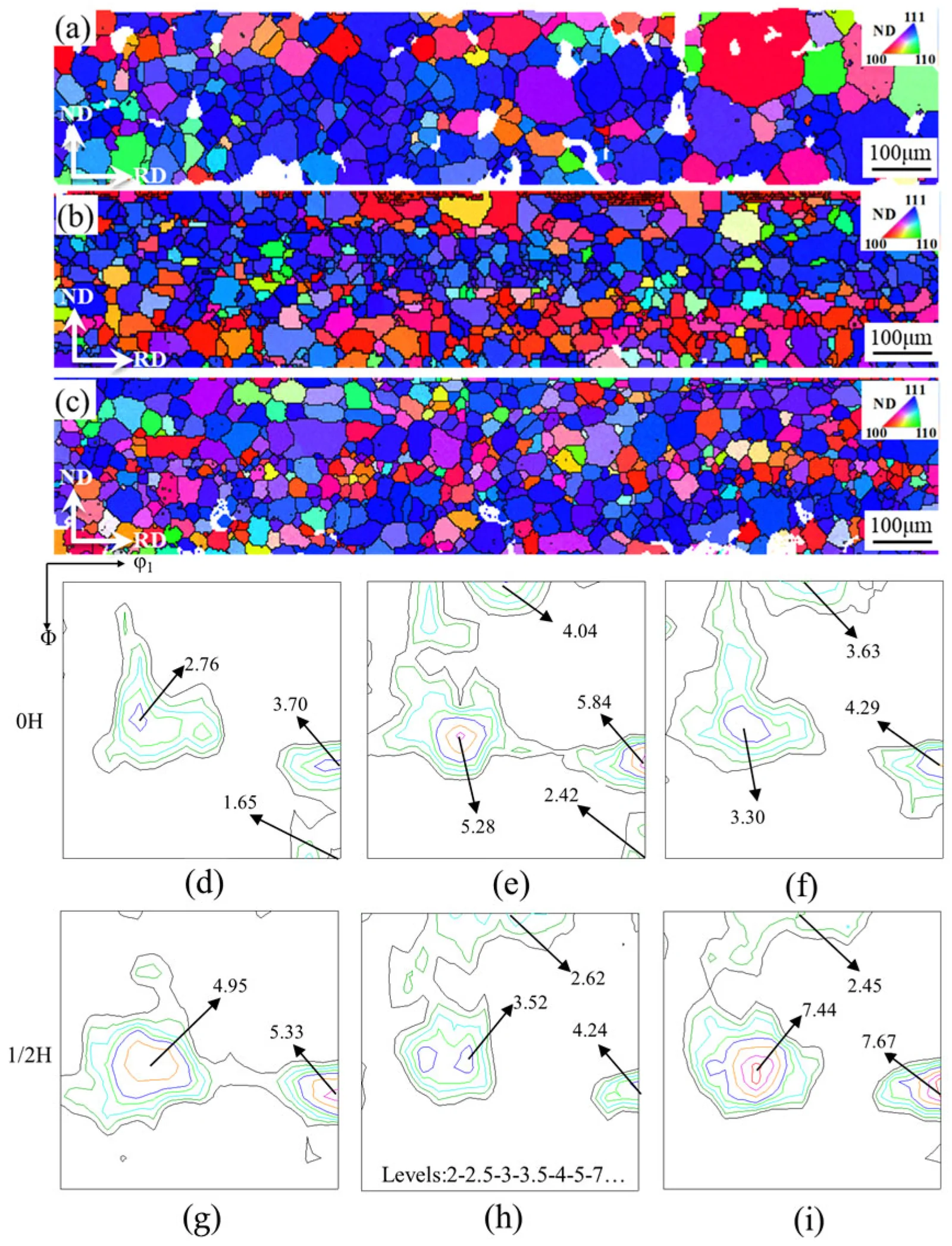
Primary-annealed microstructure and macrotexture of Fe-3 wt.% Si strips with different Y contents. (**a**) Strip A; (**b**) Strip B; (**c**) Strip C; (**d**–**f**) Surface (0H) φ_2_ = 45° ODF sections; (**g**–**i**) mid-thickness (1/2H) φ_2_ = 45° ODF sections.

**Figure 4:**
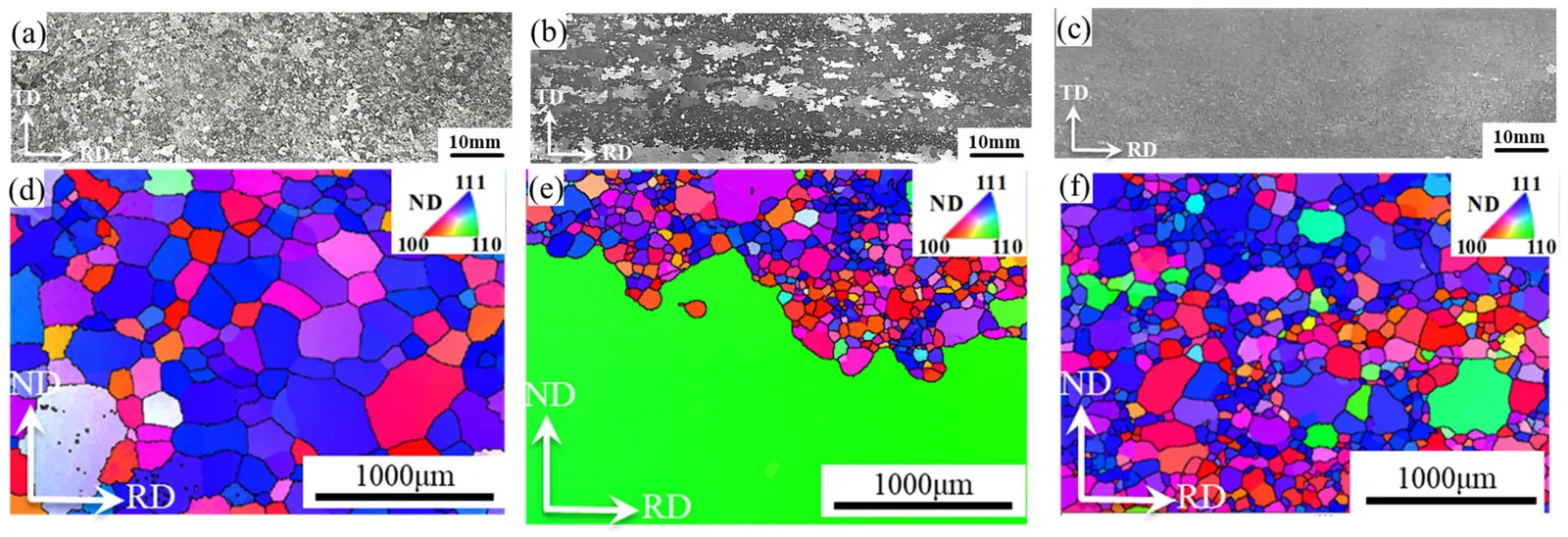
Secondary recrystallization structures of Fe-3 wt.% Si strips with different Y contents. (**a**–**c**) Macrostructures after secondary recrystallization annealing for Strips A (0 wt.% Y), B (0.05 wt.% Y), and C (0.1 wt.% Y); (**d**–**f**) RD-ND–IPF orientation maps of corresponding microstructures after secondary annealing.

**Figure 5:**
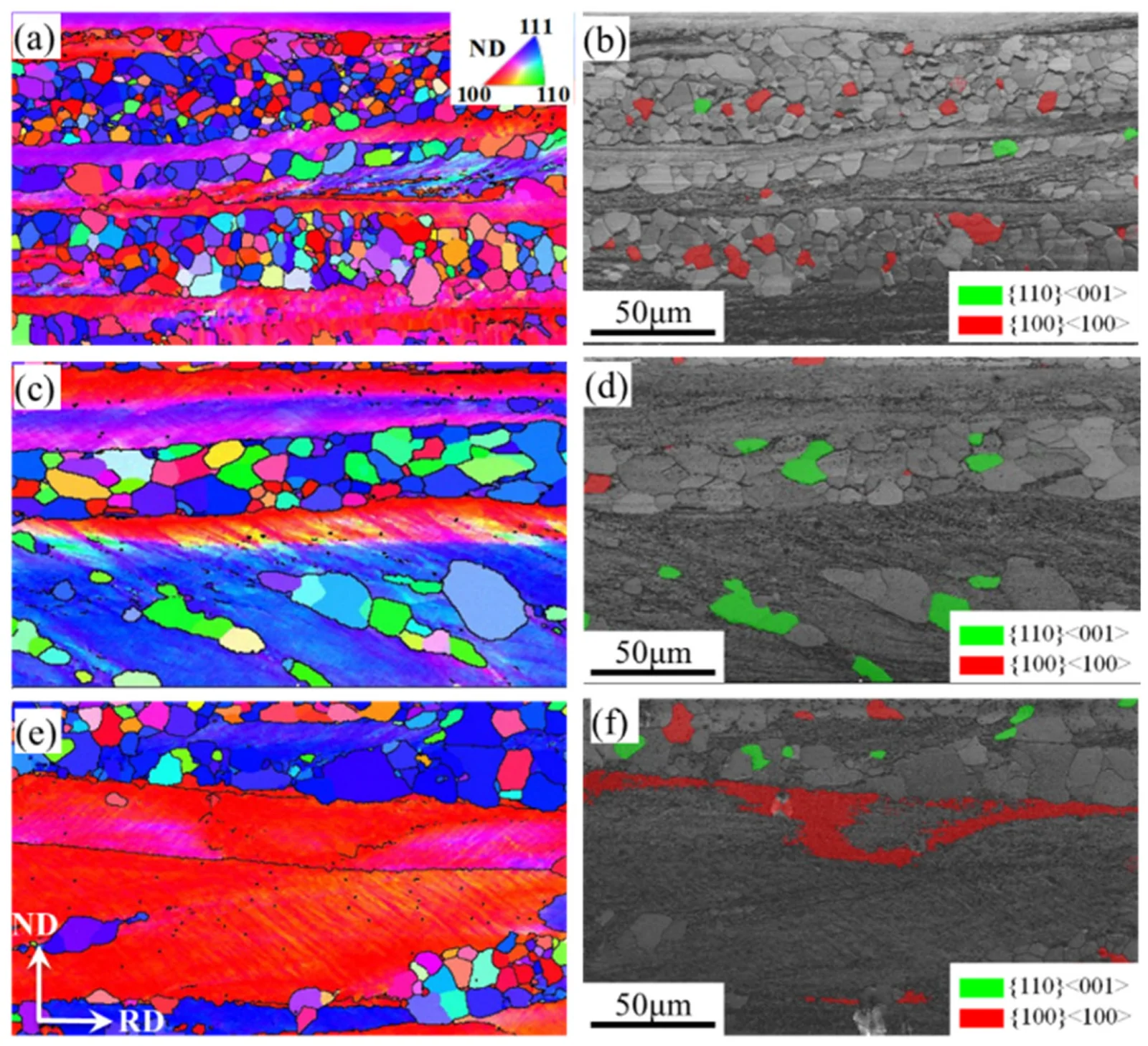
Texture distribution and shear-band-induced Goss nucleation after annealing at 800 °C for 40 s (**a**,**c**,**e**) RD-ND–IPF orientation maps of partially recrystallized sheets for Strips A (0 Y), B (0.05 wt% Y) and C (0.1 wt% Y). (**b**,**d**,**f**) Highlight maps showing Goss {110}〈001〉 in green and Cube {100}〈100〉 in red.

**Figure 6:**
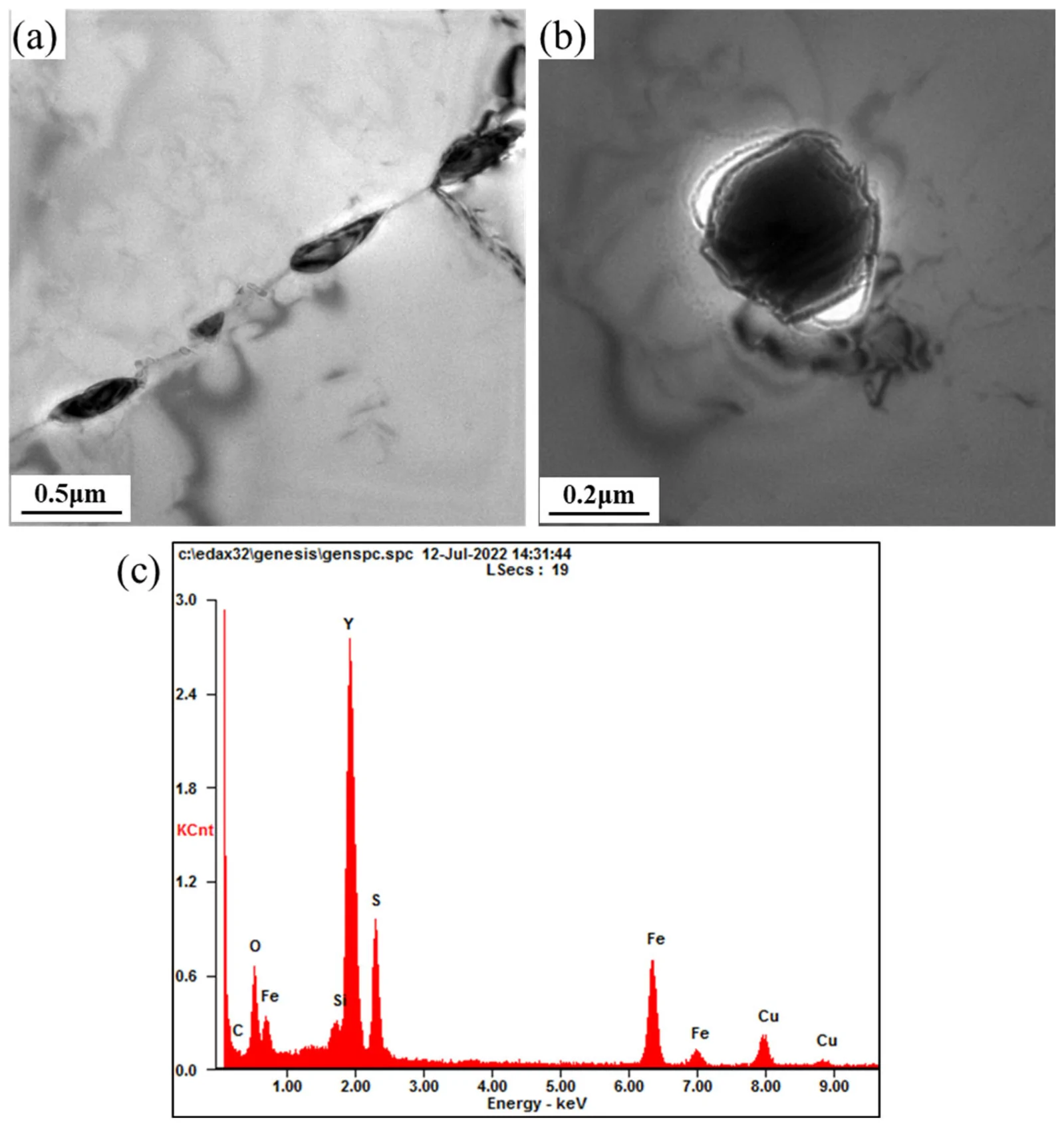
Y-containing precipitates formed under solidification conditions in Fe-3 wt.% Si-0.05 wt.% Y steel. (**a**) Precipitation at grain boundaries; (**b**) Intragranular precipitation; (**c**) EDS analysis.

**Figure 7:**
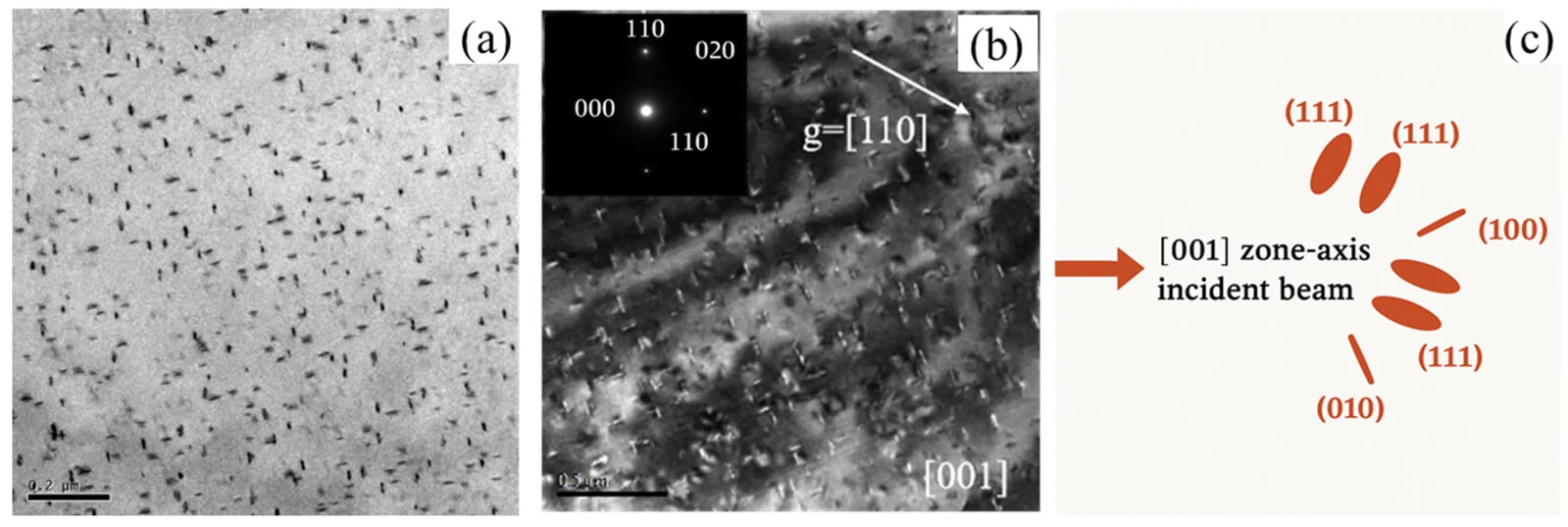
Dislocation loops in the primary recrystallization matrix of grain-oriented silicon steel. (**a**) Bright-field image under [011] zone axis; (**b**) Dark-field image under [001] zone axis; (**c**) Schematic distribution of dislocation loops with different Burgers vectors under [001] incidence (TEM results).

**Figure 8:**
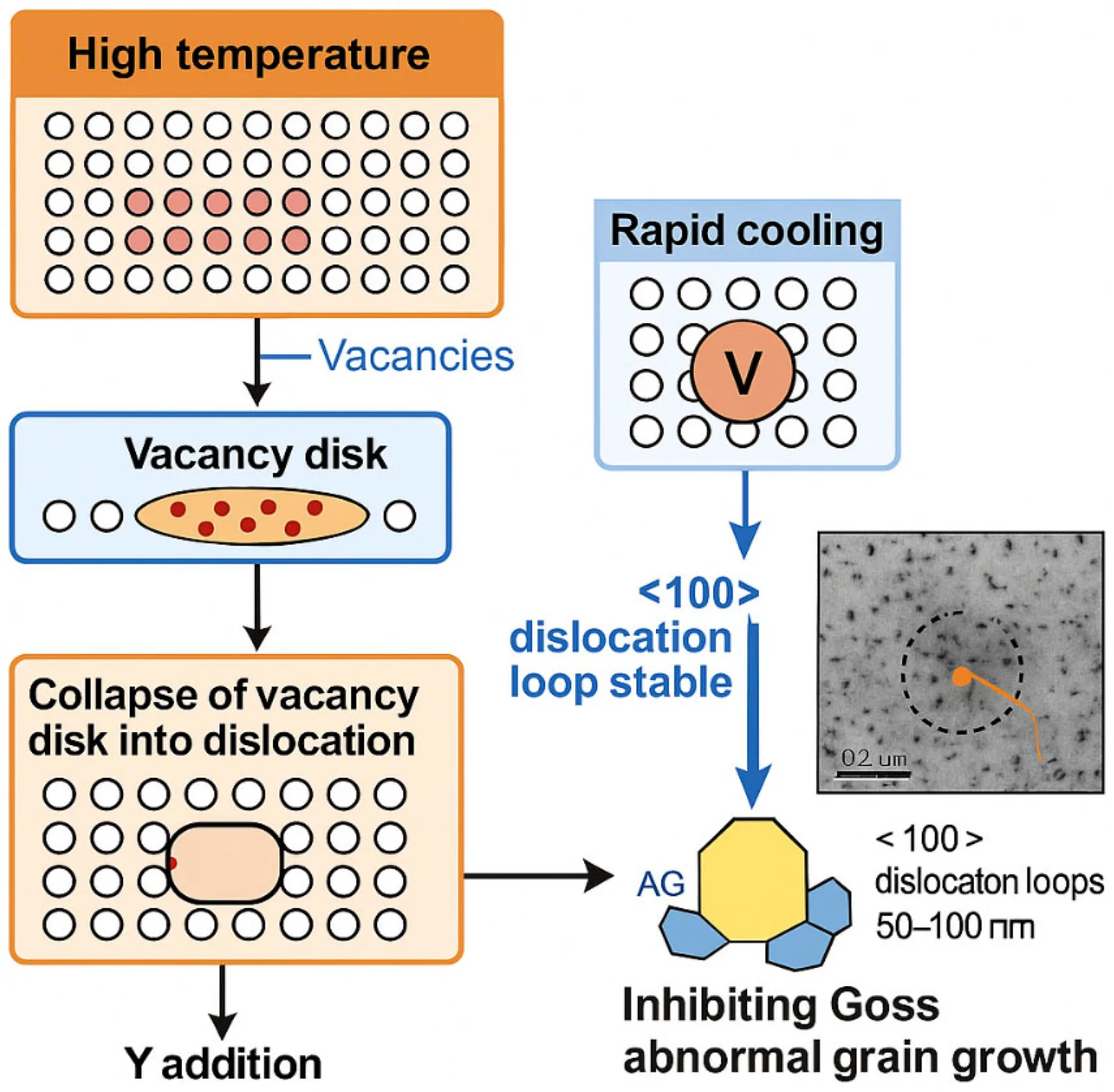
Schematic mechanism for dislocation-loop formation in silicon steel.

**Table 1:** Main chemical composition of the experimental steels (mass fraction, %).

Alloy ID	C (wt.%)	Si (wt.%)	Y (wt.%)	O (wt.%)
A	≤0.005	3.10	0	≤0.003
B	≤0.005	3.20	0.05	
C	≤0.005	3.00	0.10	

## Data Availability

The original contributions presented in this study are included in the article. Further inquiries can be directed to the corresponding authors.
